# Complete Genome Sequence of *emm*4 *Streptococcus pyogenes* MEW427, a Throat Isolate from a Child Meeting Clinical Criteria for Pediatric Autoimmune Neuropsychiatric Disorders Associated with Streptococcus (PANDAS)

**DOI:** 10.1128/genomeA.00127-16

**Published:** 2016-03-17

**Authors:** Kristin M. Jacob, Theodore Spilker, John J. LiPuma, Suzanne R. Dawid, Michael E. Watson

**Affiliations:** a Division of Pediatric Infectious Diseases, Department of Pediatrics and Communicable Diseases, University of Michigan Medical School, Ann Arbor, Michigan, USA; b Department of Microbiology and Immunology, University of Michigan Medical School, Ann Arbor, Michigan, USA

## Abstract

We report the complete genome assembly of the *Streptococcus pyogenes* type *emm*4 strain MEW427 (also referred to as strain UM001 in the Pediatric Acute-Onset Neuropsychiatric Syndrome [PANS] Research Consortium), a throat isolate from a child with acute-onset neuropsychiatric symptoms meeting clinical criteria for PANDAS (pediatric autoimmune neuropsychiatric disorders associated with streptococcus). The genome length is 1,814,455 bp with 38.51% G+C%.

## GENOME ANNOUNCEMENT

*Streptococcus pyogenes* is a Gram-positive bacterial pathogen causing a great diversity of human disease manifestations, including acute inflammatory infections and post-infectious, immune-mediated conditions (1). The clinical entity of pediatric autoimmune neuropsychiatric disorders associated with streptococcus (PANDAS) remains poorly understood. Molecular mimicry between streptococcal antigens and an associated autoimmune response to neurons in the basal ganglia and cerebral cortex is believed to cause movement disorders and acute behavioral changes seen in this condition (2, 3). We present the whole genome sequence of *S. pyogenes* MEW427 (also referenced as strain UM001 by the Pediatric Acute-Onset Neuropsychiatric Syndrome [PANS] Research Consortium), an *emm*-type 4.0 throat isolate from a female child meeting clinical criteria for PANDAS.

Chromosomal DNA of strain MEW427 was isolated using the Wizard genomic DNA purification kit (Promega, Madison, WI), and sequenced using the PacBio RS II Sequencer (Pacific Biosciences, Menlo Park, CA). Samples were prepared according to manufacturer protocols with the P6 polymerase kit and C4 sequencing reagents, with the exception of an increase to 1-h polymerase binding and 1 h binding to magnetic beads. For library construction, DNA was sheared to fragments of ~22,450 bp, isolated using the BluePippin Electrophoresis System (Sage Science, Inc., Beverly, MA). Sequences were collected using one single-molecule real-time (SMRT) cell. This generated 68,843 reads, each with a length of ~16,050 bp, for a total of 1,104.8 Mb of sequence data (~300- to 500-fold coverage). The sequence was assembled using Celera v8.3rc2 (4, 5). The resulting single scaffold was indexed and aligned against the fastq reads with BWA v0.7.12, using BWA-MEM. The resulting .sams files were sorted, indexed and converted to .bam and .bai files using SAMtools v1.2 (6, 7). Error correction was performed with Pilon v1.12 (8), and Harvest tools v1.2, employing parsnp and gingr (9). Genome overlap at the ends was identified with SeqEdit (DNASTAR, Madison, WI), and trimmed manually to position *dnaA* as the starting point. A preliminary annotation was performed using Prokka v1.11, with a reference library generated from *emm*4 strain MGAS10750 (NC_008024) (10, 11). Upon submission to GenBank the annotations were repeated using the NCBI Prokaryotic Genome Annotation Pipeline for database consistency.

The genome of *S. pyogenes* MEW427 contains 1,814,455 bp, with a 38.51% G+C% content. The number of coding regions is 1,767 with 15 rRNA and 57 tRNA genes; for comparison, strain MGAS10750 is 1,937,111 bp in length with 1,978 predicted coding regions, and 18 rRNA and 63 tRNA genes (11). The PHAge Search Tool (PHAST) identified 3 incomplete prophage regions (12). The web-based tool CRISPRFinder identified 2 candidate clustered regularly interspaced short palindromic repeat (CRISPR) regions (13). By multiple locus sequence typing (MLST), MEW427 was recognized as sequence type 39 (14). MEW427 is of type *emm*4.0 (cluster E1), a common cause of pharyngitis in North America (15). Sequence examination identified the pyrogenic exotoxins and superantigens SpeB, SpeC, SpeF, Ssa, and SmeZ. Altogether, this sequence will further inform the nature of *S. pyogenes* molecular biology and potentially help understand the pathogenesis of PANDAS.

### Nucleotide sequence accession number.

This genome sequence has been deposited in GenBank under accession number CP014138. The sequence version referred to is the first version.
